# Context-dependent roles of MDMX (MDM4) and MDM2 in breast cancer proliferation and circulating tumor cells

**DOI:** 10.1186/s13058-018-1094-8

**Published:** 2019-01-14

**Authors:** Chong Gao, Gu Xiao, Alessandra Piersigilli, Jiangtao Gou, Olorunseun Ogunwobi, Jill Bargonetti

**Affiliations:** 1 Graduate Center Biology Program, Hunter College, City University of New York, Belfer Building, New York, NY USA; 2 Department of Biological Sciences, Hunter College and Weill Cornell Medical College, City University of New York, 413 East 69th Street, Belfer Building, New York, NY 10021 USA; 3 Laboratory of Comparative Pathology, Rockefeller University, Weill Cornell Medicine and Memorial Sloan Kettering Cancer Center, New York, NY USA; 4 Department of Mathematics and Statistics, Hunter College, City University of New York, Belfer Building, New York, NY USA

**Keywords:** MDMX, MDM2, CTC, Metastasis, TNBC

## Abstract

**Introduction:**

Many human breast cancers overexpress the E3 ubiquitin ligase MDM2 and its homolog MDMX. Expression of MDM2 and MDMX occurs in estrogen receptor α-positive (ERα^+^) breast cancer and triple-negative breast cancer (TNBC). There are p53-independent influences of MDM2 and MDMX, and 80% of TNBC express mutant p53 (mtp53). MDM2 drives TNBC circulating tumor cells (CTCs) in mice, but the context-dependent influences of MDM2 and MDMX on different subtypes of breast cancers expressing mtp53 have not been determined.

**Methods:**

To assess the context-dependent roles, we carried out MDM2 and MDMX knockdown in orthotopic tumors of TNBC MDA-MB-231 cells expressing mtp53 R280K and MDM2 knockdown in ERα^+^ T47D cells expressing mtp53 L194F. The corresponding cell proliferation was scored in vitro by growth curves and in vivo by orthotopic tumor volumes. Cell migration was assessed in vitro by wound-healing assays and cell intravasation in vivo by sorting GFP-positive CTCs by flow cytometry. The metastasis gene targets were determined by an RT-PCR array card screen and verified by qRT-PCR and Western blot analysis.

**Results:**

Knocking down MDMX or MDM2 in MDA-MB-231 cells reduced cell migration and CTC detection, but only MDMX knockdown reduced tumor volumes at early time points. This is the first report of MDMX overexpression in TNBC enhancing the CTC phenotype with correlated upregulation of *CXCR4*. Experiments were carried out to compare MDM2-knockdown outcomes in nonmetastatic ERα^+^ T47D cells. The knockdown of MDM2 in ERα^+^ T47D orthotopic tumors decreased primary tumor volumes, supporting our previous finding that estrogen-activated MDM2 increases cell proliferation.

**Conclusions:**

This is the first report showing that the expression of MDM2 in ERα^+^ breast cancer and TNBC can result in different tumor-promoting outcomes. Both MDMX and MDM2 overexpression in TNBC MDA-MB-231 cells enhanced the CTC phenotype. These data indicate that both MDM2 and MDMX can promote TNBC metastasis and that it is important to consider the context-dependent roles of MDM2 family members in different subtypes of breast cancer.

**Electronic supplementary material:**

The online version of this article (10.1186/s13058-018-1094-8) contains supplementary material, which is available to authorized users.

## Introduction

The Cancer Genome Atlas has determined the molecular portraits of breast cancer, which is the second leading cause of cancer-related deaths among women [1]. It is well accepted that breast cancer is a heterogeneous disease. Five subtypes have been characterized on the basis of genes the cancers express [1]. Luminal A and B subtypes are largely estrogen receptor α (ERα)-positive and/or progesterone receptor-positive; HER2-enriched subtypes are hormone receptor-negative and HER2-positive. The basal-like and claudin-low subtypes are largely triple-negative breast cancers (TNBCs), which have none of the above markers, are associated with poor survival, and are a heterogeneous group [2]. Mutated pathways that are shared across breast cancer subtypes include mutant p53 (mtp53) and high expression of mouse double minute 2 (MDM2) [1]. In fact, 80% of TNBCs express mtp53 [1]. Increased MDM2 expression in breast cancer tissue is associated with poor prognosis [3]. MDM2 is an E3 ubiquitin ligase that targets wild-type p53 for degradation but can also act as an oncogene through p53-independent pathways (reviewed in [4]). The involvement of MDM2 in promoting breast cancer through p53-independent pathways is becoming increasingly clear. A mouse model study showed that MDM2 promotes early-stage metastasis in TNBCs, providing the first in vivo evidence for a role of MDM2 in promoting circulating tumor cells (CTCs) [5]. However, ERα^+^ breast cancer models often are not metastatic, and we and others have shown that estrogen signaling increases their cell proliferation in vitro through a p53-independent MDM2 pathway [6, 7].

The MDM2 homolog MDMX (also called MDM4) promotes breast cancer and can inhibit the transcriptional activity of p53 and promote p53 degradation by heterodimerizing with MDM2 [8, 9], but its p53-independent functions are understudied. MDMX interacts with MDM2 via the RING domain, which leads to more efficient auto-ubiquitination and degradation of both MDM2 and MDMX [10]. Haupt and colleagues analyzed the METABRIC database set [11] and found that MDMX overexpression occurs at ~ 35% in ERα^+^ luminal A and B and ~ 20% in basal breast tumors [12]. Co-occurrence of MDMX and MDM2 expression is 10% in the claudin-low subtype [12]. Therefore, more studies are needed to understand the roles of MDM2 and MDMX in promoting breast cancer phenotypes in the context of different subtypes of breast cancer.

Studies with in vitro cell culture show that MDM2 can promote cellular invasiveness by degrading E-cadherin, upregulating SNAIL protein levels, and increasing MMP9 enzymatic activity regardless of p53 mutational status [13–15]. High levels of MDMX and low levels of MDM2 have been shown to correlate with acquisition of the mesenchymal phenotype associated with metastasis of breast cancers [16]. MDMX knockdown has shown potential as a target for inhibiting the proliferation of breast cancers expressing wild-type p53 [17]. Some breast cancer cells with gain-of-function mtp53 also show an MDMX proliferative role that is mediated in part by downregulation of p27 protein levels [18]. To date, no study has been carried out to explore the role of MDMX in breast cancer metastasis. Solid tumor metastasis involves several steps, including tumor cell invasion and intravasation into the bloodstream, circulating and surviving cells in the blood, and extravasation of cells into secondary organs [19, 20].

We sought to stratify the biological functions of MDMX and MDM2 and their impacts on breast cancer development, comparing metastatic and nonmetastatic breast cancer subtypes. Using female nonobese diabetic severe combined immunodeficiency gamma (NOD.Cg-*Prkdc*^*scid*^ *Il2rg*^*tm1Wjl*^/SzJ) (NSG) immunodeficient inbred laboratory mice as the model, we assessed human breast tumor detection and development in response to MDMX or MDM2 knockdown. The tumor volume helps to assess cell viability and proliferation, whereas the number of CTCs quantitatively reflects the metastatic potential of cancer cells. We tested the role of MDM2 or MDMX knockdown in the metastatic TNBC MDA-MB-231 cells by assessing the tumor volumes and the number of endpoint CTCs. We found that MDM2 knockdown in MDA-MB-231 orthotopic tumors drastically increased MDMX protein levels and, in support of previously published data [5], also suppressed the number of CTCs. Importantly, we report, for the first time to our knowledge, that MDMX was indispensable in the metastasis cascade, because knocking down MDMX significantly blocked the presence of CTCs. Interestingly, although MDM2 or MDMX knockdown resulted in a trend toward smaller tumors, the decreases in size were only statistically significant at early time points and only with MDMX knockdown. Moreover, we identified that in primary MDA-MB-231 orthotopic tumors, there was increased expression of the human metastasis-promoting genes *CXCR4* (C-X-C chemokine receptor type 4) and *PTGS2* (prostaglandin-endoperoxide synthase 2) [21, 22]. However, the nonmetastatic ERα^+^ T47D (mtp53-expressing) orthotopic tumors showed no evidence of metastasis, but in vivo primary tumor growth was significantly decreased by the knockdown of MDM2. These findings highlight the importance of studying the MDMX and MDM2 signaling in the context of different breast cancer subtypes that express mtp53.

## Materials and methods

### Cell culture

#### 2D cell culture

Human breast cancer cell lines T47D (*mdm2* SNP309 G/G, mutant p53 L194F) and MDA-MB-231 (*mdm2* SNP309 T/G, mutant p53 R280K) were purchased from the American Type Culture Collection (www.atcc.org; Manassas, VA, USA). Cells were maintained at 5% CO_2_ in DMEM (Life Technologies, Carlsbad, CA, USA) with 50 U/ml penicillin, 50 μg/ml streptomycin (Mediatech/Corning Life Sciences, Manassas, VA, USA), and supplemented with 10% FBS (Gemini Bio-Products, West Sacramento, CA, USA) in a 37 °C humidified incubator. T47D cells generated with inducible MDM2 knockdown were described previously [6]. Constitutive MDM2 or MDMX knockdown cell lines were generated by retroviral infection with MLP.GFP vector (a generous gift from Scott Lowe) containing mir30 short hairpin RNA (shRNA)-expressing vector, *mdm2* 151656 shRNA, or *mdmx* 13023 shRNA. The mir30 shRNA inducible expressing vector has been used as a control for numerous previous high-impact studies [23, 24], and the only difference for the stable knockdown cell lines was a constitutively active promoter. Cell lines were generated and selected as previously described [7, 23]. All stable knockdown cell lines were used as selected pools.

#### 3D Matrigel culture

Cells grown in regular culture conditions were trypsinized and counted. Cells (2000 per well) were seeded on top of 40 μl of solidified Matrigel (Cultrex; Trevigen, Gaithersburg, MD, USA) in DMEM containing 10% FBS and antibiotics. Medium was replenished every 3 days.

### Cell proliferation assay

MDA-MB-231 cells (50,000/well) were seeded in a six-well plate in triplicate and were allowed to grow for 2, 4, 5, and 6 days. At each time point, cells were trypsinized, and the number of cells was determined by cell counting using a hemocytometer.

### Wound-healing assay

Cells (800,000/well) were plated in a six-well plate one night before the experiment. Scratches were created using a 200-μl pipette tip. Cells were then rinsed three times with fresh medium. Wound closure was observed within the scrape line and photographed by phase-contrast microscopy. Wound area was measured and quantified by using NIS-Elements software (Nikon Instruments, Melville, NY, USA). Thirty fields per condition were recorded, and three independent experiments were performed. Transient electroporation of small interfering RNA (siRNA) was carried out using an Invitrogen Neon transfection system (Life Technologies) with ON-TARGET siRNA smartpools obtained from Dharmacon (Lafayette, CO, USA): siGENOME™ Control Pool (catalogue no. D-001206-13-20), human *mdm2* siRNA (catalogue no. L-003279-00), and human *mdm4* siRNA (catalogue no. L-006536-02-0005).

### RNA isolation, real-time qRT-PCR, and microarray analysis

RNA was extracted using QIAshredder columns and RNeasy Mini Kit (Qiagen, Hilden, Germany) following manufacturer’s protocol. Complementary DNA (cDNA) synthesis was carried out using the High-Capacity cDNA Archive Kit reagents (Applied Biosystems, Foster City, CA, USA). RT Master Mix and RNA were mixed and incubated at 25 °C for 10 min and then at 37 °C for 2 h. Amplification of gene transcripts was performed by qPCR with primer probes for *mdm2*(3–4) (Hs01069930_m1), *mdmx* (Hs00910358_s1), *cxcr4* (Hs00607978_s1), *ptgs2* (Hs00153133_m1), and *gapdh* (Ha02758991_g1) from Applied Biosystems. Primers were combined with 150 ng of cDNA and TaqMan Universal Master Mix (Applied Biosystems), and reactions were carried out using the standard program in the QuantStudio 7 sequence detection system (Applied Biosystems). cDNA (25 ng) from tumor samples was used in TaqMan™ Array Human Tumor Metastasis (Applied Biosystems) following the manufacturer’s protocol. The gene expression analysis was performed with ExpressionSuite software (Thermo Fisher Scientific, Waltham, MA, USA).

### Protein extraction

Cells were harvested at 1100 rpm for 5 min at 4 °C, then washed three times with ice-cold PBS. For extraction from tissues, samples were snap-frozen in liquid nitrogen and homogenized. Cells then were resuspended in radioimmunoprecipitation assay buffer (0.1% SDS, 1% IGEPAL Nonidet P-40, 0.5% deoxycholate, 150 mM NaCl, 1 mM ethylenediaminetetraacetic acid, 0.5 mM ethylene glycol-bis(β-aminoethyl ether)-*N,N,N′,N′*-tetraacetic acid, 50 mM Tris-HCl, pH 8.0, 1 mM phenylmethylsulfonyl fluoride, 8.5 μg/ml aprotinin, and 2 μg/ml leupeptin). The cell suspension was incubated on ice for 30 min to lyse the cells, vortexing occasionally. Additional sonication of lysate three times for 30 s/30 s rest on ice at 98% amplitude was done after the incubation. Samples were centrifuged at 13,000 rpm for 30 min at 4 °C.

### Immunoblotting assay

4× NuPAGE lithium dodecyl sulfate buffer (Life Technologies) and 20 mM dithiothreitol (DTT) were added to protein extracts, and samples were heated at 70 °C for 10 min. Iodoacetamide (100 mM; MilliporeSigma, Burlington, MA, USA) was then added to the samples when cooled down. For CXCR4 detection, extracts were incubated with the same buffer containing DTT and iodoacetamide at room temperature for 20 min. Ten percent SDS-PAGE or 4–12% gradient SDS-PAGE (Life Technologies) was used to separate samples, followed by electrotransfer onto nitrocellulose membrane or polyvinylidene fluoride membrane. The membrane was blocked with 5% nonfat milk (Bio-Rad Laboratories, Hercules, CA, USA) in either 1× PBS with 0.1% Tween 20 or 1× Tris-buffered saline TBS with 0.1% Tween 20 following incubation of primary antibody overnight at 4 °C. The next day, the membrane was washed with either 1× PBS with 0.1% Tween 20 or 1× TBS with 0.1% Tween 20 and then incubated with secondary antibody for 1 h at room temperature. Signal was detected by chemiluminescence with a Pierce Super Signal Kit (Thermo Fisher Scientific) and autoradiographed with HyBlot CL films (Thomas Scientific, Swedesboro, NJ, USA).

### Immunofluorescence

Cells were grown in 3D culture conditions described above. After 8 days of culturing, colonies were washed with 1× PBS and fixed with 4% paraformaldehyde (MilliporeSigma) for 15 min at room temperature. The plates were washed three times with 1× PBS, permeabilized with 0.5% Triton X-100 in PBS/1% FBS for 10 min and incubated with rhodamine-phalloidin (BK005; Cytoskeleton, Denver, CO, USA) for 1 h at room temperature. Alexa Fluor-conjugated secondary antibody (Life Technologies) was used, and cells were mounted with VECTASHIELD mounting medium (Vector Laboratories, Burlingame, CA, USA) containing 4′,6-diamidino-2-phenylindole (DAPI). Images were captured with a Nikon A1 confocal microscope at 200× magnification and analyzed by NIS-Elements AR Analysis software (Nikon Instruments).

### Antibodies

Antibodies used were MDM2 (1:1:1 mix of mouse monoclonal 4B2, 2A9, 4B11 hybridoma supernatant), p53 (1:1:1 mix of mouse monoclonal 240,421,1801 hybridoma supernatant), and MDMX (Proteintech, Rosemont, IL, USA), actin-HRP (Santa Cruz Biotechnology, Dallas, TX, USA), E-cadherin (Cell Signaling Technology, Danvers, MA, USA), and CXCR4 (Abcam, Cambridge, MA, USA).

### Orthotopic tumor implantation and measurement

For MDA-MB-231 study, 1 × 10^7^ cells with constitutive MDM2 or MDMX knockdown were injected into the mammary fat pad of female NSG mice at 6 weeks of age. No additional drug was administered. Tumor growth was measured using calipers, and tumor volume was calculated as volume = π/6 (length × width × width). At ethical endpoint, mice were killed following institute guidelines. For T47D study, MDM2 knockdown was induced with 4 μg/ml doxycycline in cell culture conditions for 10 days before implantation. Tumor cells (1 × 10^7^) were then injected into the mammary fat pad of female NSG mice at 6 weeks of age. Animals were provided with drinking water containing 2 mg/ml doxycycline (MilliporeSigma) dissolved in deionized water, 8 μg/ml 17β-estradiol (MilliporeSigma) dissolved in DMSO, and 2% sucrose (MilliporeSigma), replenished every other day.

### Circulating tumor cell analysis

Cardiac punctures were performed at the endpoint of the experiment, and blood samples were stored temporarily in 1.5-ml microcentrifuge tubes coated with sodium heparin (Sagent Pharmaceuticals, Schaumburg, IL, USA) prior to CTC isolation procedure. Briefly, whole blood was subjected to centrifugation. After removal of plasma, the buffy coat layers were then collected and subjected to red blood cell (RBC) lysis to remove residual RBCs. Flow cytometric analysis was performed using a FACScan device (BD Biosciences, San Jose, CA, USA), and event counting was gated on the basis of size and GFP intensity from cultured cells as positive controls. The number of CTCs was obtained by dividing the number of positive events by individual blood volume. Statistical significance was calculated by two-sample permutation test, two-sided hypothesis after multiplicity adjustment (Hochberg procedure).

### Tissue processing and histology

Animal tissues were harvested, fixed in 10% buffered formalin, and embedded in paraffin. Sections of primary tumors and lungs were cut at 5 μm and stained with H&E by the Laboratory of Comparative Pathology. The slides were analyzed by a board-certified veterinary pathologist (AP).

### Statistics

CTC data obtained from the MDA-MB-231 animal study were analyzed using R statistical software (version 3.4.2; R Foundation for Statistical Computing, Vienna, Austria). Datasets were tested for assumptions of normality using the Shapiro-Wilk test [25]. If the normality was confirmed, a pairwise independent *t* test was carried out. Otherwise, for nonnormal data, we applied a permutation-based two-sample *t* test instead, which is appropriate for small samples from nonnormal distributions. Permutation tests were performed using DAAG (data analysis and graphics) version 1.22 in the R package. Hochberg correction [26, 27] was performed on the resulting *p* values for all multiple comparisons to control for the familywise error rate [28]. All other graphs and statistical analysis were generated using Prism 7.01 software (GraphPad Software, La Jolla, CA, USA). In the box-and-whisker plots, each dot represents one mouse.

## Results

### MDM2 and MDMX potentiate release of MDA-MB-231 circulating tumor cells

**Fig. 1:**
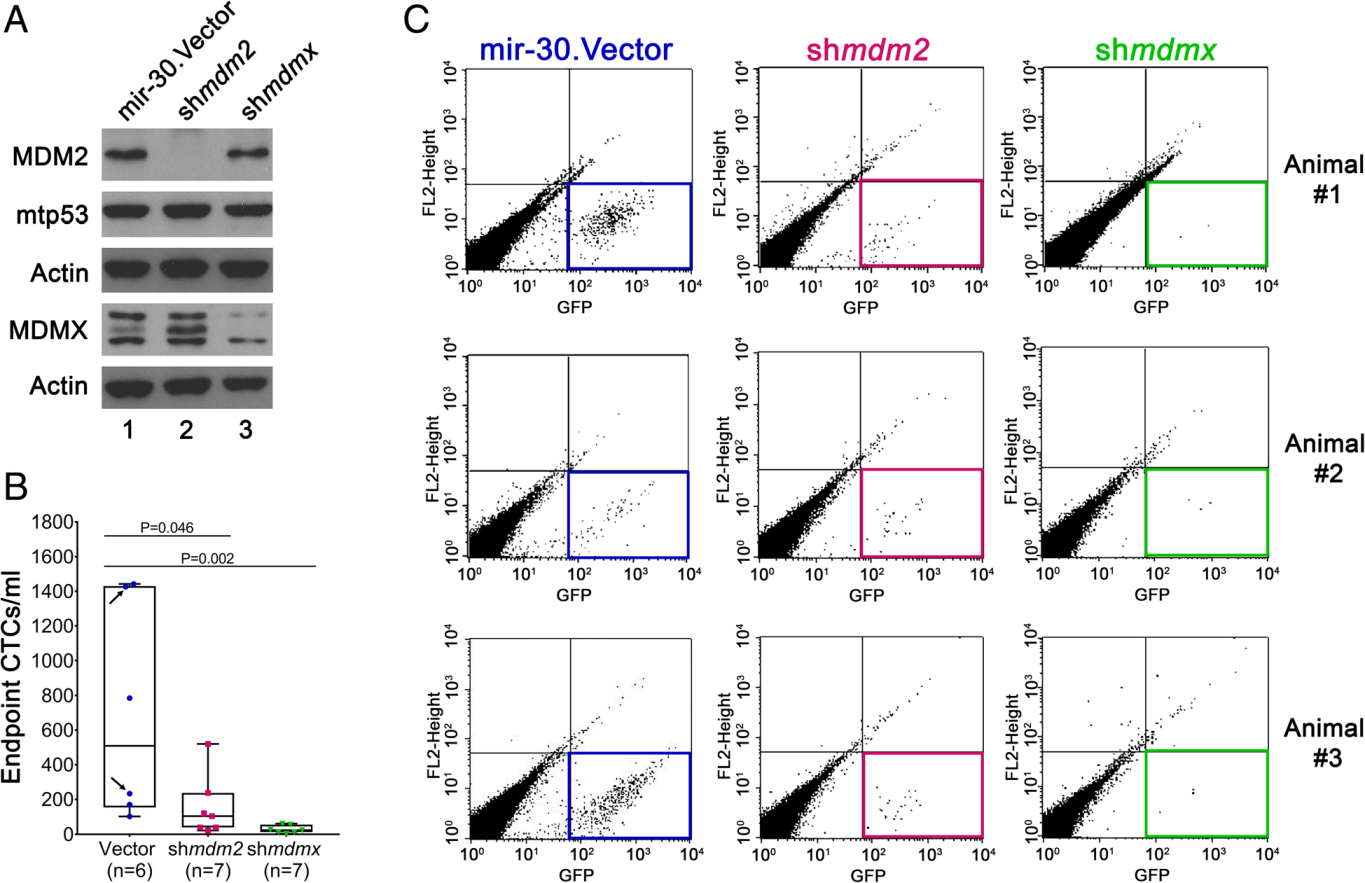
MDMX and MDM2 knockdown in MDA-MB-231 orthotopic transplants reduces CTCs. MDA-MB-231 cells with constitutive sh*mdm2*, sh*mdmx*, or mir30 shRNA-expressing vector were implanted into the mammary fat pads of 6-week-old female NSG mice. **a** Western blot analysis of MDM2, MDMX, and mtp53 protein levels from 50 μg of whole-cell lysates from 231.mir30.vector, 231.sh*mdm2*, and 231.sh*mdmx* cells (lanes 1, 2, and 3, respectively) prior to mammary fat pad implantation. Actin is shown as a loading control. **b** Box-and-whisker plot represents the numbers of CTCs per milliliter from 231.mir30.vector, 231.sh*mdm2*, and 231.sh*mdmx* cells engrafted into animals. The number of CTCs was determined by flow cytometry, and the total events were counted (gates were set by the GFP signal intensity and cell size). The number of CTCs per milliliter was obtained by dividing the number of positive events by blood volume from individual animals. The adjusted *p* value was obtained with two-tailed, two-sample *t* tests using a permutation test. **c** Representative fluorescence-activated cell sorting plots showing GFP-positive events in different mouse groups

MDM2 promotes early-stage metastasis and CTCs in TNBC [5], but the role of MDMX has not been defined. To better understand the role of MDM2 and MDMX in breast cancer metastasis, we compared the biological outcomes of knocking them down in the highly metastatic triple-negative claudin-low MDA-MB-231 cells. The MDA-MB-231 cells carry a p53 R280K mutation, *mdm2*, with mRNA overexpression due to heterozygous SNP309 T/G and elevated MDMX expression [18]. We first asked if MDM2 or MDMX modulated the early-stage metastasis in an orthotopic NSG mouse model by examining how their genetic knockdown influenced the number of CTCs. We generated isogenic MDA-MB-231 cell lines with constitutive MDM2 or MDMX knockdown and mir30 shRNA-expressing vector controls that could easily be scored by GFP expression. Western blot analysis showed a significant continuous reduction of MDM2 or MDMX prior to implanting the cells into the mouse model (Fig. 1a). MDM2 depletion increased MDMX protein levels in cell culture, indicating that MDM2 functioned as an E3 ligase toward MDMX (Fig. 1a). To determine if the knockdown of MDM2 or MDMX expression decreased the CTCs, we scored the number of GFP-positive cell counts per milliliter of blood at the endpoint of the experiment. The NSG mouse model is well documented for the study of breast cancer metastasis [29]. When mir30 shRNA-expressing vector control MDA-MB-231 cells were implanted into mice, they generated an average of 693 CTCs per milliliter of blood (Fig. 1b). Stable MDM2 knockdown in MDA-MB-231.sh*mdm2* cells resulted in a 78% reduction of CTCs (155 cells/ml), and MDMX knockdown in stable MDA-MB-231.sh*mdmx* knockdown decreased the CTCs to 29 cells/ml, which was a staggering 96% reduction (Fig. 1b, c). This study supports the finding that MDM2 promotes TNBC CTCs [5] and is the first study to report that MDMX promotes TNBC CTCs.

### MDMX expression in MDA-MB-231 cells moderately influences tumor growth

**Fig. 2:**
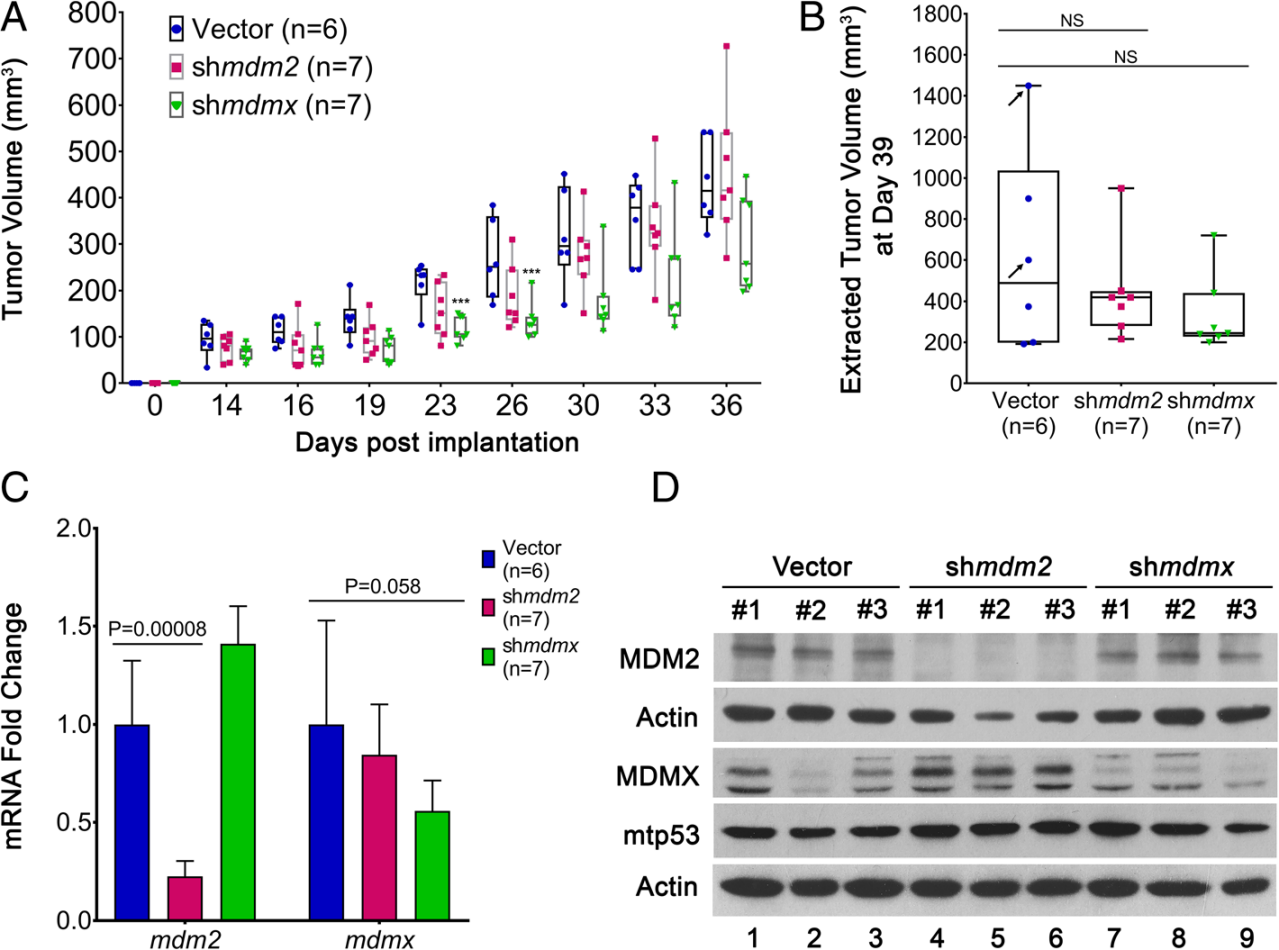
MDMX and MDM2 knockdown in MDA-MB-231 orthotopic transplants does not significantly reduce primary tumor growth. **a** Primary tumor volumes of 231.mir30.vector (*n* = 6), 231.sh*mdm2* (*n* = 7), and 231.sh*mdmx* (n = 7) engrafted animals were measured using calipers over 36 days. **b** The endpoint tumor volumes were determined on dissected masses at the time of necropsy. **c** mRNA levels of *mdm2* and *mdmx* normalized to *gapdh* in primary tumors were determined by real-time qRT-PCR. Error bars represent SD. * *p* < 0.05, ** *p* < 0.01, *** *p* < 0.001, NS = nonsignificant. The *p* value was calculated using two-tailed unpaired *t* tests. **d** Protein expression of MDM2, MDMX, and mtp53 from 231.mir30.vector, 231.sh*mdm2*, and 231.sh*mdmx* engrafted primary tumors were determined by Western blot analysis. Three tumors per group were used, and actin is shown as a loading control

We found an increased number of CTCs when MDA-MB-231 cancer cells expressed high levels of both MDM2 and MDMX. We investigated whether the reduced CTCs correlated with knockdown of MDM2 or MDMX might be a result of reduced primary tumor size at any point during the tumor development. Published results with knockdown of MDM2 in tumor-derived MDA-MB-231 cells showed an inconclusive influence of MDM2 on tumor volume with no difference in the documented final weights [5]. We found that depletion of MDM2 resulted in a non–statistically significant reduction in the average primary tumor volume at all points during the experiment (Fig. 2a and b). MDMX knockdown, on the other hand, caused a statistically significant smaller tumor volume at the early stage of measurements (up to day 26), but at the experimental endpoint there was no statistically significant difference (Fig. 2a and b). Additionally, the primary tumors analyzed at the experimental endpoint exhibited similar local invasion in histopathological analysis, regardless of MDM2 or MDMX knockdown (Additional file 1: Figure S1). The histopathology of the lungs of all mice revealed the presence of metastases. The metastatic burden (number, size) appeared rather heterogeneous in the different groups, with some animals displaying only few and small groups of neoplastic cells. However, on digital slides, a semiquantitative assessment of the tumor burden in the mice indicated that MDM2 knockdown and MDMX knockdown reduced metastasis (Additional file 2: Figure S2).

We confirmed in vivo knockdown of MDMX and MDM2 from the tumors using qRT-PCR and Western blot analysis (Fig. 2c and d). Significant depletion of MDM2 in the tumor tissue was detected, and we also detected an increase in MDMX protein (Fig. 2c and d, lanes 4, 5, and 6). The shRNA-mediated decrease in *mdmx* mRNA was clear but had an insignificant *p* value resulting from two animals from the mir30 vector control group with random loss of *mdmx* expression (Fig. 2c). These random loss endpoint tumors corresponded to the largest and intermediate-sized masses (marked by arrows in Fig. 2b). The statistical conclusions were the same with or without the two random loss animals. Importantly, animals with clear MDMX protein depletion showed no associated change in MDM2 (Fig. 2d).

### MDMX and MDM2 knockdown decrease cell migration in vitro for MDA-MB-231 cells

**Fig. 3:**
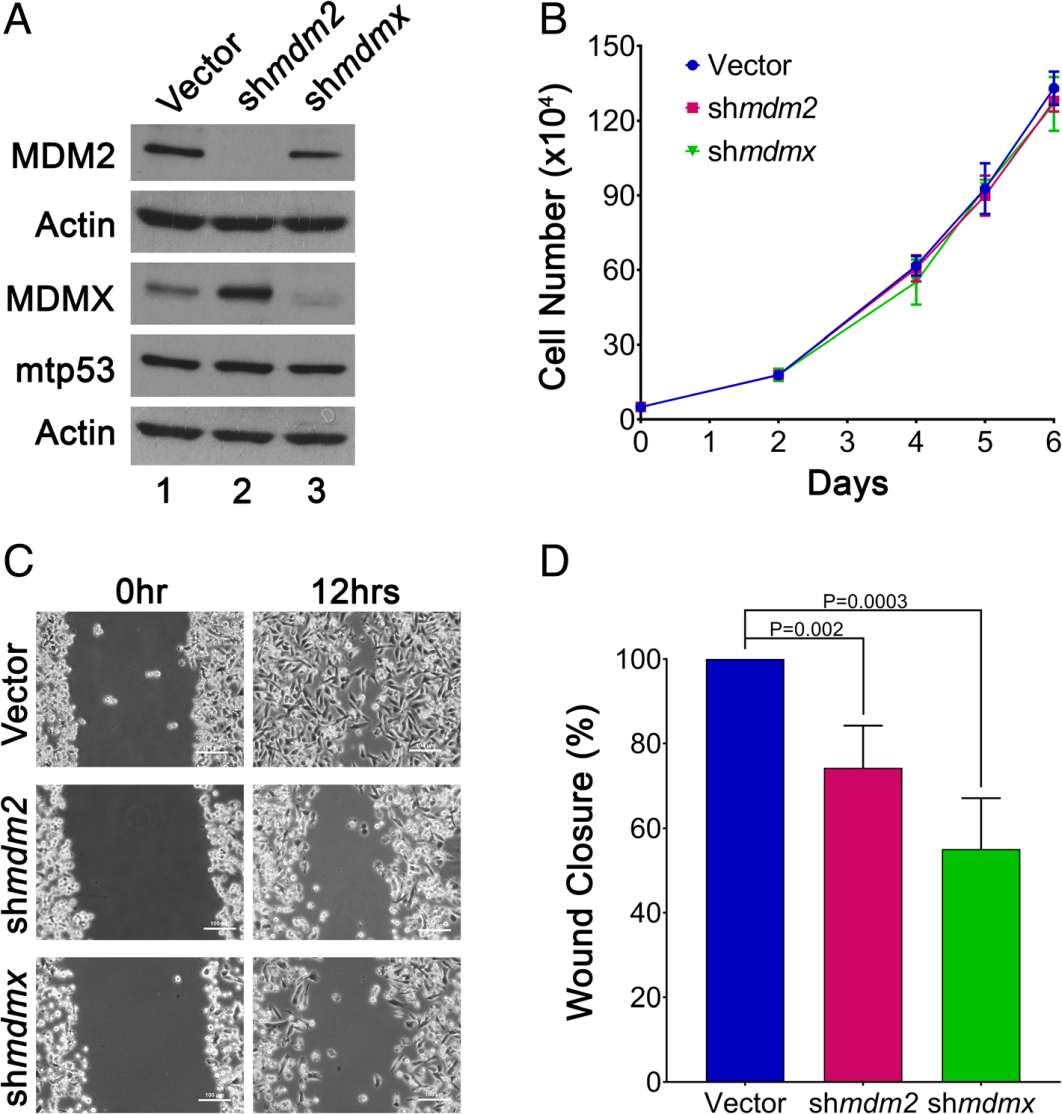
MDMX and MDM2 provoke in vitro MDA-MB-231 cell migration without altering cell proliferation. **a** Representative Western blot demonstrating the levels of MDM2, MDMX, and mtp53 in 231.mir30.vector, 231.sh*mdm2*, and 231.sh*mdmx* cells (lanes 1, 2, and 3, respectively). Fifty micrograms of lysate was loaded per lane. Actin was used as a loading control. **b** The number of cells was determined by hemocytometer cell counting. Cells (*n* = 50,000) were seeded in triplicate, and cell counting was performed at 2, 4, 5, and 6 days. Dots represent mean values, and error bars represent SD. Experiments were carried out with three biological replicates. **c** Wound closure was observed by phase-contrast microscopy and photographed at 0 and 12 h. One representative image from each group at 0 and 12 h is shown. **d** The wound area was measured by using NIS-Elements software (Nikon Instruments, Melville, NY, USA). The percentage of wound closure was quantified from four independent biological experiments. The *p* value was obtained by two-tailed unpaired *t* test

We previously showed that inducible knockdown of MDM2 has no influence on MDA-MB-231 cell proliferation or viability [7]. In the present study, we used 2D and 3D cell culture systems to determine whether stable knockdown of either MDMX or MDM2 influenced the MDA-MB-231 cell proliferation or migration properties. The cell proliferation was not changed by either MDM2 or MDMX knockdown in 2D cell culture (Fig. 3a and b). In keeping with the ability of increased MDM2 and MDMX to promote CTCs, we saw that MDM2 and MDMX knockdown reduced the in vitro cell migration by 30% and 50%, respectively (Fig. 3c and d). We observed, as previously reported, extremely disordered and invasive morphology when MDA-MB-231 cells were grown in laminin-rich Matrigel [30]. Constitutive knockdown of MDMX in 3D Matrigel condition resulted in less disordered morphology, coupled with smaller colony sizes after 8 days, but MDM2 knockdown produced a more disordered morphology (Additional file 3: Figure S3). This observation resembled the higher penetrance of MDMX than with MDM2 knockdown on reduced tumor volume at the early stage of tumor development. Through multiple assays, we observed that both MDMX and MDM2 in claudin-low/triple-negative MDA-MB-231 cells promoted migration, and MDMX provided a moderate proliferative advantage. We also tested the influence of MDM2 and MDMX by knockdown with transient electroporation using siRNA targeting in both MDA-MB-468 cells and MDA-MB-231 cells (Additional file 4: Figure S4). Both cell lines showed reduced migration following either MDM2 or MDMX siRNA-mediated knockdown (Additional file 4: Figure S4). Our data presented in Figs. 1, 2, and 3 and Additional file 4: Figure S4 support published work using an alternative MDM2-knockdown method in MDA-MB-231 and MDA-MB-468 cells demonstrating that MDM2 promotes cell migration and CTCs [5].

### MDMX knockdown in TNBC tumors decreases transcription of *CXCR4* and *PTGS2*

**Fig. 4:**
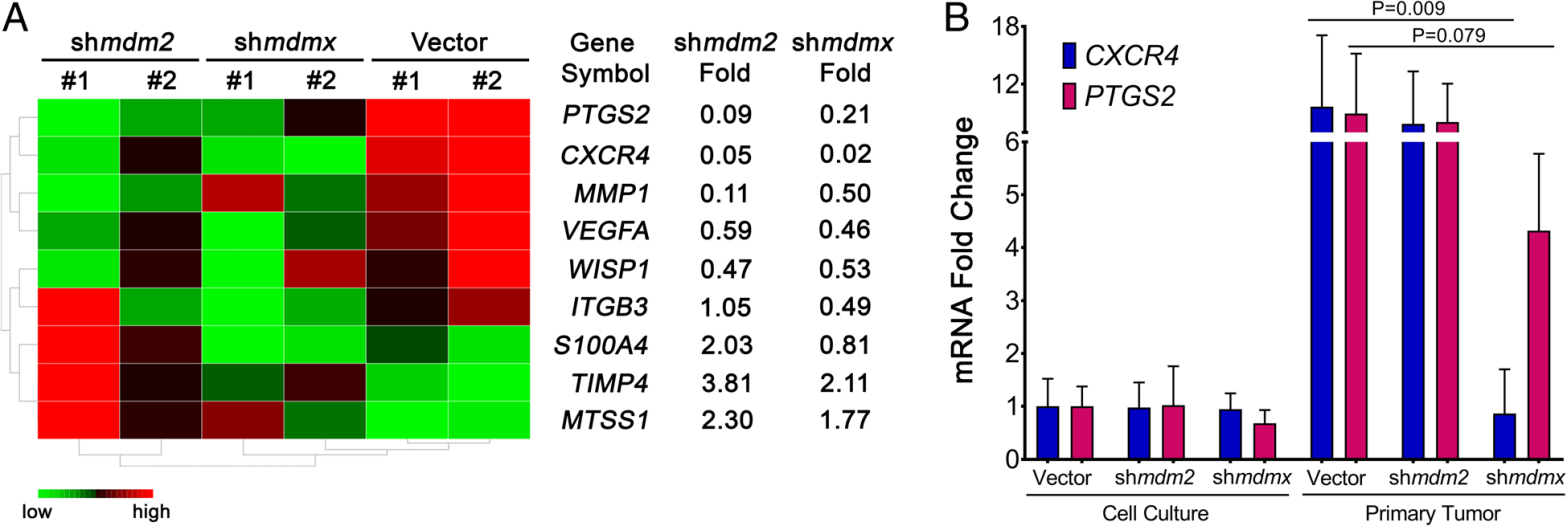
MDMX knockdown in primary tumors blocks the transcription of *CXCR4* and *PTGS2*. The 231.mir30.vector-, 231.sh*mdm2*-, and 231.sh*mdmx*-derived primary tumors were lysed and used for total RNA extraction and complementary DNA synthesis. **a** Microarray analysis revealed selected tumor metastasis-related genes that were either up- or downregulated in 231.sh*mdm2* and 231.sh*mdmx* compared with 231.mir30 vector. Fold changes were gated either > 2 or < 0.5. Two tumor samples per group were used for the analysis. **b** From the respective cells derived from all the primary tumors, the total *CXCR4* and *PTGS2* levels were determined by real-time qRT-PCR, and these were compared with those of the parental cells grown in culture. The bars represent mean values, and error bars represent SD. The *p* values were obtained by two-tailed unpaired *t* test

We explored metastasis targets using a predesigned RT-PCR microarray card screened with RNA prepared from the orthotopic NSG tumor tissues from MDA-MB-231.mir30.vector, MDA-MB-231.sh*mdm2*, and MDA-MB-231.sh*mdmx* cells. The predesigned human metastasis microarrays carry primers to 88 tumor suppressors or oncogenes. We identified a subset of gene expression targets positively and negatively regulated by MDM2 or MDMX knockdown. We assembled the targets into three clusters with fold change thresholds set below 0.5 or above 2. The top hits are displayed in a heat map, with the most notably positively associated genes being *PTGS2* and *CXCR4* (Fig. 4a). Importantly, *CXCR4* and *PTGS2* are established as genes that mediate organ-specific metastasis in TNBCs [20, 31]. *CXCR4* encodes a G protein-coupled receptor protein that binds to CXCL12 ligand and is highly expressed in patients with breast cancer [32]. The CXCR4 pathway has been implicated in many types of cancer and enhances cell proliferation, increases cell survival, and enhances invasion and metastasis (reviewed in [33]). PTGS2/COX2 is involved in the prostaglandin 17β-estradiol (E2) pathway and promotes breast cancer progression [34, 35]. Expression of both *CXCR4* and *PTGS2* promotes MDA-MB-231 lung metastasis [21]. We validated the downregulation of *PTGS2* and *CXCR4* with MDM2 and MDMX knockdown in the tumors by performing qRT-PCR from the sets of primary tumor samples. The tissues showed MDMX knockdown to be associated with a 95% downregulation of *CXCR4* transcripts and a 65% downregulation of *PTGS2* (Fig. 4b). Both *PTGS2* and *CXCR4* showed an MDM2-associated reduction, but the MDM2 knockdown-associated changes were not statistically significant. We compared the *CXCR4* and *PTGS2* expression in the tumors with the expression in the starting cell lines and observed a dramatic increase in tumor tissue for all samples except the MDMX knockdown cells. We also observed that although MDMX knockdown decreased *CXCR4* in tumors, it did not do so in cell culture (Fig. 4b). MDM2 and MDMX promote metastasis, but only MDMX knockdown correlated with a reduction in *CXCR4* expression. It is unclear what this means at this time.

### MDM2 facilitates ERα^+^ T47D xenograft primary tumor growth

**Fig. 5:**
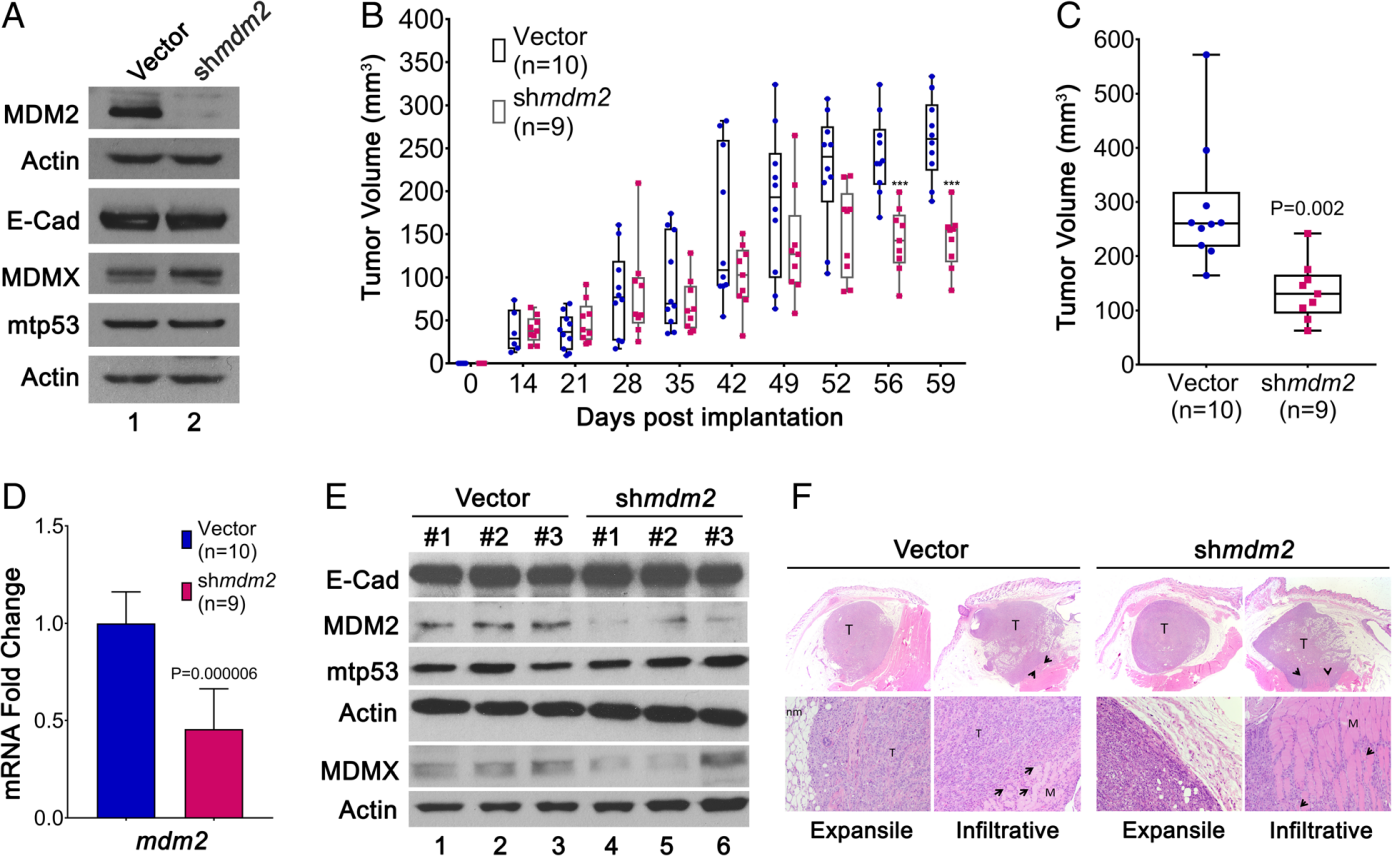
MDM2 knockdown in ERα^+^ T47D orthotopic transplant reduces tumor volume. **a** T47D cells with inducible sh*mdm2* or mir30 shRNA-expressing control vector were treated with 4 μg/ml doxycycline (Dox) for 10 days to induce and maintain shRNA expression. Western blot shows the levels of MDM2, MDMX, E-cadherin, and mtp53 with Dox treatment (lanes 1 and 2) prior to mammary fat pat implantation. Actin was used as a loading control. **b** Animals were provided with 2 mg/ml Dox and 8 μg/ml E_2_ in their drinking water during the entire experiment. Primary tumor growth was measured over a period of 60 days using calipers *** *p* < 0.001 calculated by two-tailed unpaired *t* test. **c** The experimental endpoint tumor volume was determined at the time of necropsy. **d** *mdm2* mRNA expression in primary tumors was determined by real-time qRT-PCR. The *p* value was determined by two-tailed unpaired *t* test. **e** E-cadherin, MDM2, MDMX, and mtp53 protein levels from primary tumors were determined by Western blot analysis. Actin was used as the loading control. **f** Representative H&E staining images of T47D.vector and T47D.sh*mdm2* under 200× and 1000× magnification. T represents Tumor; nm represents normal mammary fat pad; M represents muscle; arrowhead depicts tumor cells infiltrating into muscle layer

We previously showed that MDM2 provides an estrogen-mediated proliferative advantage to breast cancer cells and disrupts acini formation by increasing phosphorylation of Rb and elevating E2F1 protein levels [7]. We used the estrogen receptor α-positive (ERα^+^)/MDM2 and mtp53-overexpressing breast cancer cell line T47D to test this relationship in the NSG orthotopic mouse model. T47D cells with or without induced shRNA-mediated MDM2 knockdown were implanted into the mammary fat pads of mice. These nonmetastatic cells, as expected, did not generate CTCs (data not shown). Therefore, the context of cancer subtype influences the experimental outcome with respect to the roles of MDM2 family members. In the ERα^+^ context for T47D cells, metastasis outcomes did not occur at the endpoints we tested, but we were able to ask how MDM2 expression influenced the tumor volume. Consistent with our previous findings [6, 7], when MDM2 was knocked down, no change was observed for mtp53 protein levels (Fig. 5a). There was also no change in E-cadherin levels (Fig. 5a). Importantly, we detected slower tumor growth in the MDM2 knockdown group than in the vector control group (Fig. 5b). A 50% reduction in the final tumor volume was confirmed after animals were killed, indicating that MDM2 drives estrogen-mediated ERα^+^ breast cancer cell proliferation in vivo (Fig. 5b and c). We confirmed the downregulation of MDM2 in the tumors by qRT-PCR and Western blot analyses (Fig. 5d and e). *Mdm2* RNA was reduced by 55%, and its protein levels were also significantly reduced. The histopathology of the primary tumors showed no differences in local invasiveness (Fig. 5f). Penetration of vessels within or adjacent to the primary mass was occasionally seen in the different groups (Fig. 5f). Additionally, the E-cadherin protein expression in primary MDM2 knockdown tumors remained unchanged. Our data showed that MDM2 promotes in vivo proliferation of ERα^+^ T47D cells without influencing invasive properties.

### Expression of MDM2, MDMX, and CXCR4 in the context of ERα^+^ versus TNBC tumors

**Fig. 6:**
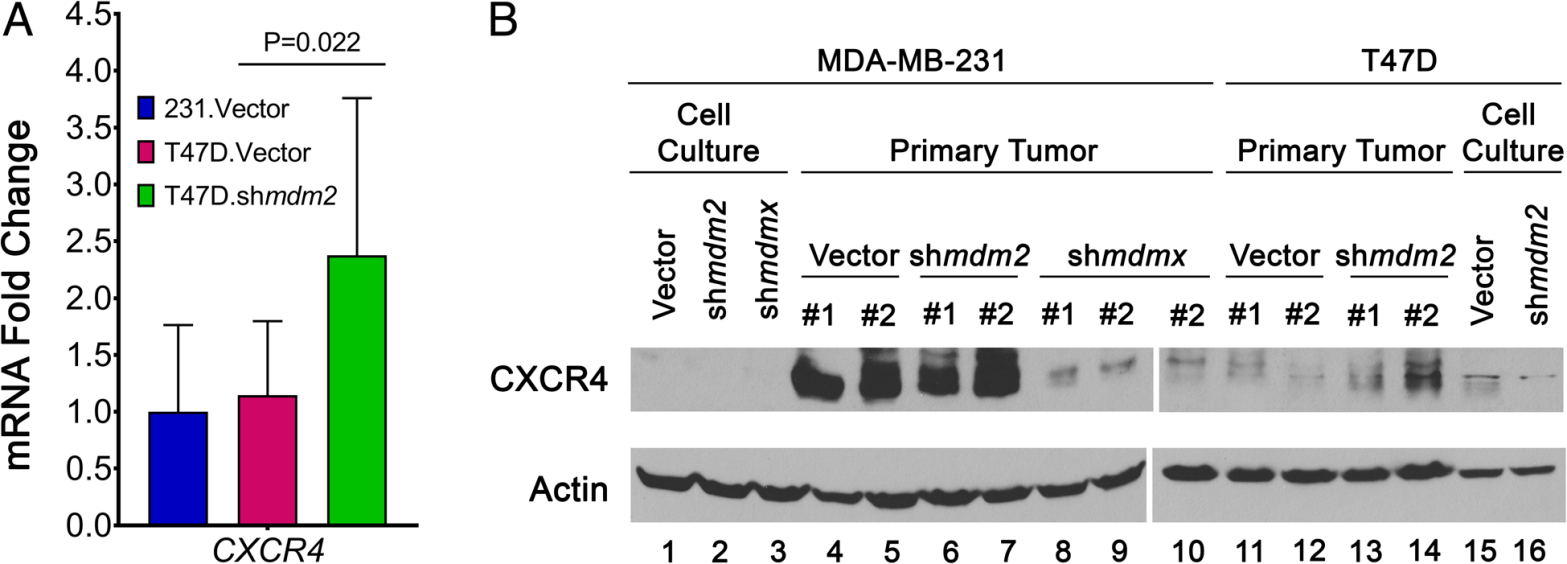
Comparative levels of CXCR4 in ERα^+^ T47D and TNBC MDA-MB-231 tumors. **a** *CXCR4* RNA expression normalized to *gapdh* with MDM2 knockdown was determined using real-time qRT-PCR. 231.mir30.vector (*n* = 6), T47D.mir30.vector (*n* = 10), and T47D.sh*mdm2* (*n* = 9) tumor samples were analyzed. The RNA level of *CXCR4* was set as 1 for the 231.mir30.vector group, and T47D samples were expressed relative to 231.mir30.vector values. **b** Protein expression of CXCR4 was compared from parental cell lines and two tumors from each group. Representative Western blot demonstrating protein levels of CXCR4 and actin in MDA-MB-231 and T47D groups shown using two gels (lanes 1–9 and 10–16, with tumor 2 for 231.sh*mdmx* used in lane 10 as a common reference)

In this study, we compared different subtypes of breast cancer cells, and we were interested in the comparative expression of MDM2, MDMX, and CXCR4 in the different contexts. We observed that T47D and MDA-MB-231 cells had similar levels of cytoplasmic MDM2 and MDMX, but T47D cells had more of both proteins on the chromatin (Additional file 5: Figure S5). The decreased MDA-MB-231 *CXCR4* expression in MDMX knockdown tumors correlated with the reduction of tumor metastasis for MDA-MB-231 cells, but MDM2 knockdown reduced MDA-MB-231 metastasis without a correlated reduction in *CXCR4*. Furthermore, because we did not see a change in *CXCR4* or *PTGS2* in the cell lines following MDM2 or MDMX knockdown, we reasoned that these genes were not direct targets and were only targets in the context of the animal model (Fig. 4b). We therefore asked if the orthotopic T47D tumors demonstrated any changes in their *CXCR4* expression (Fig. 6a). The T47D.vector *CXCR4* average mRNA level (from ten tumors) was the same as that observed for the MDA-MB-231.mir30.vector tumor samples (from six tumors). Additionally, the T47D.sh*mdm2* tumors with decreased MDM2 had a statistically significant increase in *CXCR4* mRNA (Fig. 6a). We tried to compare the relative CXCR4 protein expression in the T47D and MDA-MB-231 tumors by observing a few tumors from each set. This gave variable outcomes, and we observed that the tumors from MDA-MB-231.mir30.vector, MDA-MB-231.sh*mdm2*, MDA-MB-231.sh*mdmx*, T47D.mir30.vector, and T47D.sh*mdm2* showed highly variable CXCR4 protein levels. What is clear is first that *mdmx* knockdown tumors displayed reduced CXCR4 protein levels compared with vector control or *mdm2* knockdown tumors (which was consistent with our mRNA analysis) and second that the context of cell type (and if the cells were grown in the animal or on a culture dish) changed the regulation of CXCR4 (Fig. 6b). The function of MDM2 might be different between the two cell lines in part because one is metastatic and the other is not. More studies will be needed to clarify the complex relationship that exists for stroma signaling to tumor in the context of breast cancer subtypes.

## Discussion

MDMX and MDM2 are expressed in multiple subtypes of breast cancer [1]. MDMX and MDM2 overexpression promote tumorigenic potential through blocking p53 and also through p53-independent influences [4, 36–38]. Mouse models addressing the p53-independent influence of MDM2 overexpression in mammary gland tumorigenesis show that p53^−/−^ transgenic mice with MDM2 overexpression have an increased incidence of tumorigenesis [39]. The role of MDMX overexpression in tumorigenesis appears to vary dependent on the system being studied. In one mouse model, the MDMX transgene increases mammary tumor development and enhances tumor development in a heterozygous mutant p53 or a p53-null background [40, 41]. However, in an alternative mouse model, overexpression of homozygous MDMX transgenes results in embryonic lethality, whereas the hemizygous animals are viable and do not have accelerated tumor formation [42]. Mouse models vary and do not always recapitulate human disease. In the orthotopic model in the present study, both MDM2 and MDMX significantly enhanced the metastatic potential of the MDA-MB-231 cells, but they did not significantly increase the final tumor volume (Figs. 1 and 2).

**Fig. 7:**
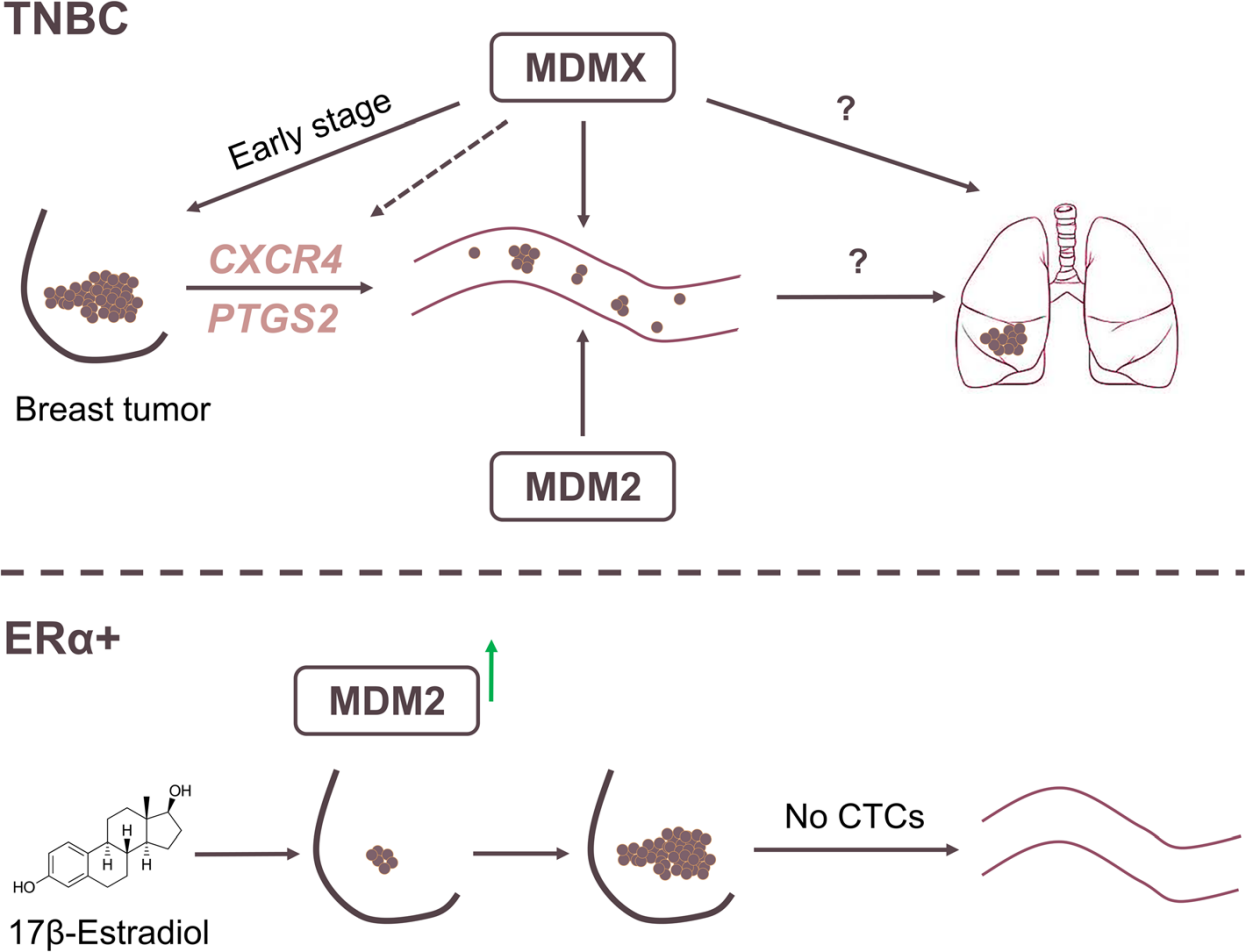
MDMX and MDM2 in TNBC promote metastasis, and in ERα^+^ breast cancer MDM2 promotes proliferation. In TNBC, MDMX promotes expression of *CXCR4* and *PTGS2* with associated release of CTCs but no increase in cell proliferation. In ERα^+^ breast cancer, estrogen stimulates MDM2 expression with no influence on *CXCR4* and causes an increase in cell proliferation without correlated metastasis

Hauck and colleagues demonstrated that MDM2 is required for the promotion of mtp53-independent TNBC metastasis [5]. In the present study, we confirmed that MDM2 provokes CTC formation from TNBC and report for the first time that MDMX expression also plays an active role in the production of breast cancer CTCs. The examination of CTCs is one component of liquid biopsy [43]. Our discovery that MDMX robustly promotes breast cancer CTCs has implications for breast cancer liquid biopsy using MDMX as a biomarker. It will be important in the future to investigate whether the differences in CTCs from breast cancers with or without MDMX correlates with the ability of cells to seed and recolonize in secondary sites (Fig. 7 model).

In this report, we also uncovered *CXCR4* and *PTGS2* as two key target genes modulated by MDMX in primary tumors but not when the cells are grown in the culture dish. Interestingly, although MDM2 promotes the cancer cell release from the primary tumor into the circulating system, the observed downregulation of *CXCR4* or *PTGS2* expression by MDM2 knockdown was not statistically significant (Fig. 4b). One possible explanation for this is that when MDM2 was depleted, there was a consistent sharp increase in MDMX protein levels. This high MDMX in turn could upregulate *CXCR4* and *PTGS2*. The increased MDMX upon MDM2 depletion may compensate for the depletion of MDM2. MDMX knockdown did not increase MDM2 levels, which could explain the significant result observed for decreased CTC release upon MDMX knockdown and the strongly observed inhibition of *CXCR4* and *PTGS2* transcription. Further experiments in the context of the microenvironment are required to explore this model.

Elevated *CXCR4* expression has been documented in more than 23 different types of cancers with various origins and has been shown as a poor prognostic biomarker [44]. *CXCR4* overexpression in breast cancer has been shown to promote metastasis in an organ-specific manner, and new treatments targeting this pathway in TNBC have had some success [45]. Inhibition of CXCR4 protein also leads to significantly less metastatic burden in mouse models [22, 46, 47]. It is known that in the tumor microenvironment, inflammation plays a significant role in activating CXCR4 signaling [48]. In oral squamous cell carcinoma and glioblastoma, vascular endothelial growth factor has been shown to upregulate *CXCR4* expression [49, 50]. Additionally, induction of *CXCR4* and *PTGS2* can be achieved through activation of NF-κB signaling [51]. MDM2 modulates NF-κB signaling by directly inducing the transcription of *p65* and increasing *p100* transcripts, independently of p53 [52, 53]. However, there has been no investigation on deciphering the role of MDMX in relation to metastasis-promoting pathways. It is conceivable that inflammation and/or angiogenesis in the tumor microenvironment contributes to the activation of *CXCR4* and *PTGS2* in MDMX-overexpressing breast tumor cells. We documented that MDMX correlated with transcriptional activation of *CXCR4* and *PTGS2* in primary orthotopic tumors; however, the in vitro cell culture system expressed tenfold lower *CXCR4* and *PTGS2* transcripts that were unchanged by MDMX expression. This indicates that the tumor microenvironment provides stimulatory signals that activate the pathways. It is unclear what specific cue(s) in the tumor niche define(s) the activation in our model system and, more important, what role MDMX plays in facilitating and/or maintaining such induction.

In the TNBC cells in this preclinical mouse model, we observed a reduction in tumor volume only during early time point measurements when MDMX was knocked down. Thus, targeting MDM2 and MDMX in TNBCs may have more benefit for diagnosis through liquid biopsy and for targeting metastatic disease, rather than in treating patients’ primary tumors. Targeting MDM2 and MDMX may provide therapeutic value for patients with advanced stages of TNBC. In nonmetastatic ERα^+^ T47D breast cancer cells, we generated in vivo evidence that MDM2 promoted tumor growth in response to estrogen signaling without promoting tumor-invasive properties. Therefore, targeting MDM2 has promise for targeting primary ERα^+^ tumors. New studies suggest that an excellent strategy will be to combine treatments that block MDMX and MDM2 [12, 18]. Such combination trials will have potential positive benefits for all subtypes of breast cancer.

Importantly, our results showed that high levels of MDMX and MDM2 promoted a metastatic phenotype that correlated with increased CTCs and tumors expressing increased levels of *CXCR4* and *PTGS2*. We found that MDMX had a strong influence on promoting CTCs, and upregulating *CXCR4* and *PTGS2*. The fact that both *CXCR4* and *PTGS2* have been identified as key mediators of breast cancer metastasis to bone and lung [21, 22] provides a potential new combination targeting approach coupling MDM2, MDMX, CXCR4, and PTGS2 inhibition for a mechanism to block breast cancer metastasis.

## Conclusions

Our findings provide novel insights into the roles of MDM2 and MDMX promoting CTCs of TNBC. We also documented that MDM2 promotes tumorigenesis of ERα^+^ breast cancers. Importantly, we discovered that MDMX correlates with the increased transcription of *CXCR4* and *PTGS2* in tumor tissue. Our observation that MDMX and MDM2 signaling pathways are different in TNBC and ERα^+^ breast cells has set the stage for suggesting the use of these biomarkers to more accurately define the nature of breast cancer subtypes.

## Additional files

Additional file 1: **Figure S1.** MDA-MB-231 transplants have locally invasive growth. H&E staining of three representative images from 231.mir30.vector, 231.sh*mdm2* and 231.sh*mdmx* derived primary tumors (A) at 12.5X and (B) at 200X magnification. All tumors had a locally invasive growth at the orthotopic transplantation site. T represents the primary tumor, M represents the muscle and nm represents normal mammary fat pad. (JPG 10407 kb)

Additional file 2: **Figure S2.** MDA-MB-231 cells implanted into animals display metastatic burden in the lungs that is reduced by MDM2 or MDMX knockdown. Representative images of metastatic burden in the lungs in vector control group. A) Shows representative comparisons of lungs from animals with 231.mir30.vector, 231.sh*mdm2* or 231.sh*mdmx* engrafted cells. Arrow points to metastases. H&E staining at 12.5X (upper panel) and 200X (lower panel) magnification. B) Shows a quantitation of the lung metastasis. (JPG 9785 kb)

Additional file 3: **Figure S3.** MDMX silencing leads to a less metastatic phenotype and smaller colony size in 3D culture. MDA-MB-231 cells from 231.mir30.vector, 231.sh*mdm2* and 231.sh*mdmx* were cultured in Matrigel for 8 days with medium being supplemented every 3 days. Colonies were then fixed and stained for DAPI/nuclei and F-Actin. (A) Two representative confocal images with maximal projection per group are shown. Images were taken under 200X magnification. (B) Percent of area occupied by colonies was measured and quantified by pixel intensity using NIS-Elements software. Results were quantified from two independent experiments with 30–60 colonies per group analyzed each time. (JPG 3318 kb)

Additional file 4: **Figure S4.** siRNA-mediated MDM2 or MDMX silencing reduced MDA-MB-231 and MDA-MB-468 cell migration. (A-C) MDA-MB-231 cells and (D-F) MDA-MB-468 cells. The wound closure with compared with siRNA control, si*mdm2*, or si*mdmx* and 50 μg of lysates were loaded per lane for validation of the knockdown. Actin was used as loading control. Wound closure was observed by phase-contrast microscopy and photographed at 0 and 12 h. One representative image from each group at 0 and 12 h for MDA-MB-231 cells and 0 and 24 h for MDA-MB-468 cells. One representative image from each group at the abovementioned time points is shown. The wound area was measured by NIS-Elements software. The percentage of wound closure was quantified from two independent biological experiments. The *p*-value was obtained with two-tailed unpaired *t*-test. (G-I) Shows results from stable mir30 expressing selected MDA-MB-468 cell lines and the percentage of wound closure was quantified from three independent biological experiments. (TIF 35939 kb)

Additional file 5: **Figure S5.** Variable Levels of MDM2 and MDMX in Different Breast Cancer Cell Lines. Cell lysates from fractionated samples were analyzed to compare the relative levels of MDM2 and MDMX. Lanes 1–4 show cytoplasmic and 5–8 show chromatin proteins as indicated. Only ERα ^+^ cell lines MCF-7 and T47D showed high levels of chromatin localized MDM2 (this correlated with the activation of cell proliferation by MDM2). Fractionation was carried out as previously described [54]. (TIF 4685 kb)

Additional file 6: Gene expression profile from human metastasis microarray. (CSV 26 kb)

## Abbreviations

cDNA: Complementary DNA
CTC: Circulating tumor cell
CXCR4: C-X-C chemokine receptor type 4
DAPI: 4′,6-Diamidino-2-phenylindole
DOX: Doxycycline
DTT: Dithiothreitol
E2: 17β-Estradiol
ERα: Estrogen receptor α
MDM2: Mouse double minute 2
MDMX: Mouse double minute x
NSG: Nonobese diabetic severe combined immunodeficiency gamma mouse, NOD.Cg-*Prkdc*^*scid*^ *Il2rg*^*tm1Wjl*^/SzJ
PTGS2: Prostaglandin-endoperoxide synthase 2
RBC: Red blood cell
shRNA: Short hairpin RNA
siRNA: Small interfering RNA
TBS: Tris-buffered saline
TNBC: Triple-negative breast cancer
