# Supplementary figures and images for: Context-dependent roles of MDMX (MDM4) and MDM2 in breast cancer proliferation and circulating tumor cells
Source: Breast Cancer Res. 2019 Jan 14;21:5. doi: 10.1186/s13058-018-1094-8 (PMC6332579; doi:10.1186/s13058-018-1094-8)

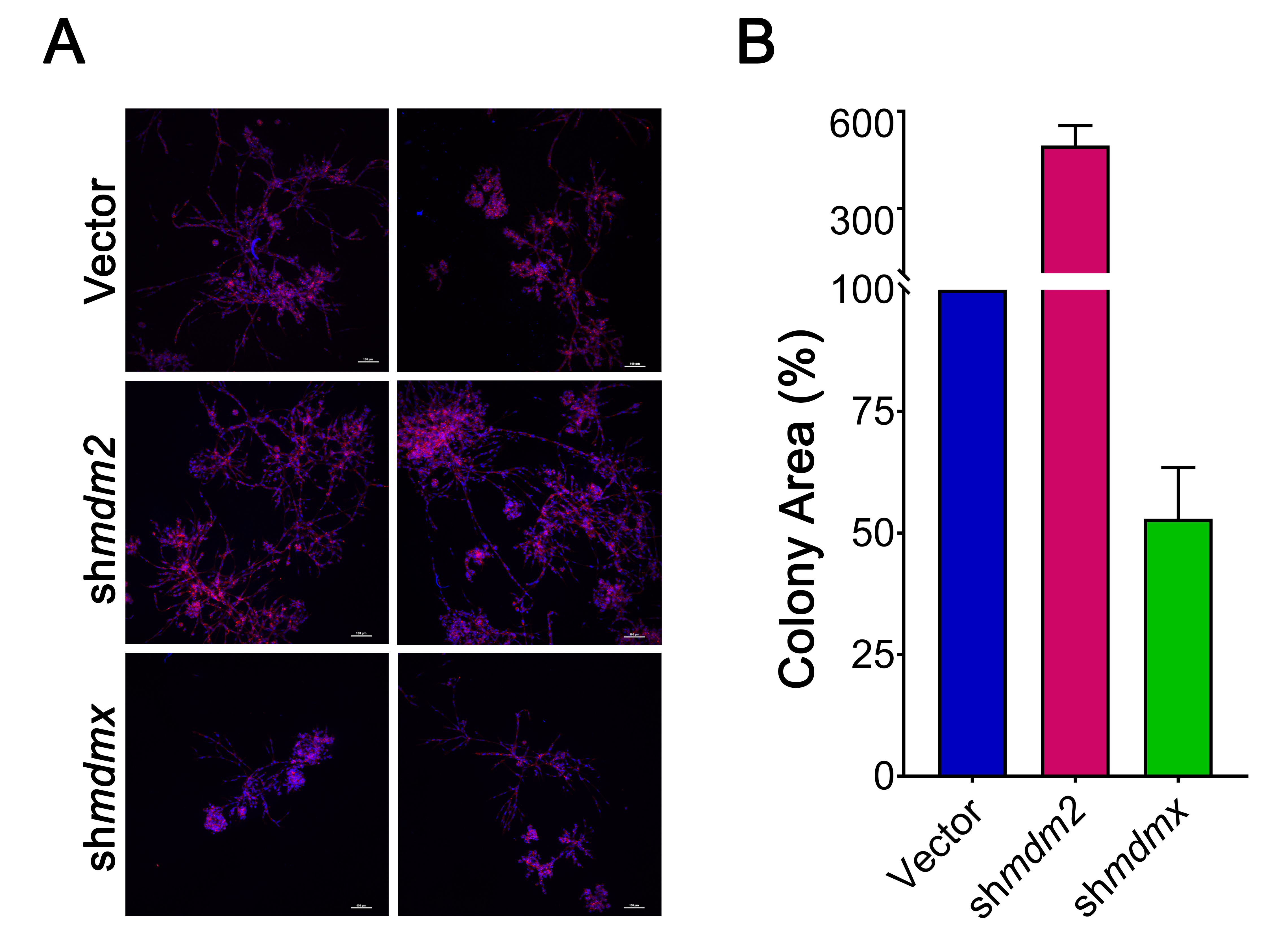

Supplement: Supplementary file 3 — Figure S3. MDMX silencing leads to a less metastatic phenotype and smaller colony size in 3D culture. MDA-MB-231 cells from 231.mir30.vector, 231.shmdm2 and 231.shmdmx were cultured in Matrigel for 8 days with medium being supplemented every 3 days. Colonies were then fixed and stained for DAPI/nuclei and F-Actin. (A) Two representative confocal images with maximal projection per group are shown. Images were taken under 200X magnification. (B) Percent of area occupied by colonies was measured and quantified by pixel intensity using NIS-Elements software. Results were quantified from two independent experiments with 30–60 colonies per group analyzed each time. (JPG 3318 kb) [file 13058_2018_1094_MOESM3_ESM.jpg]

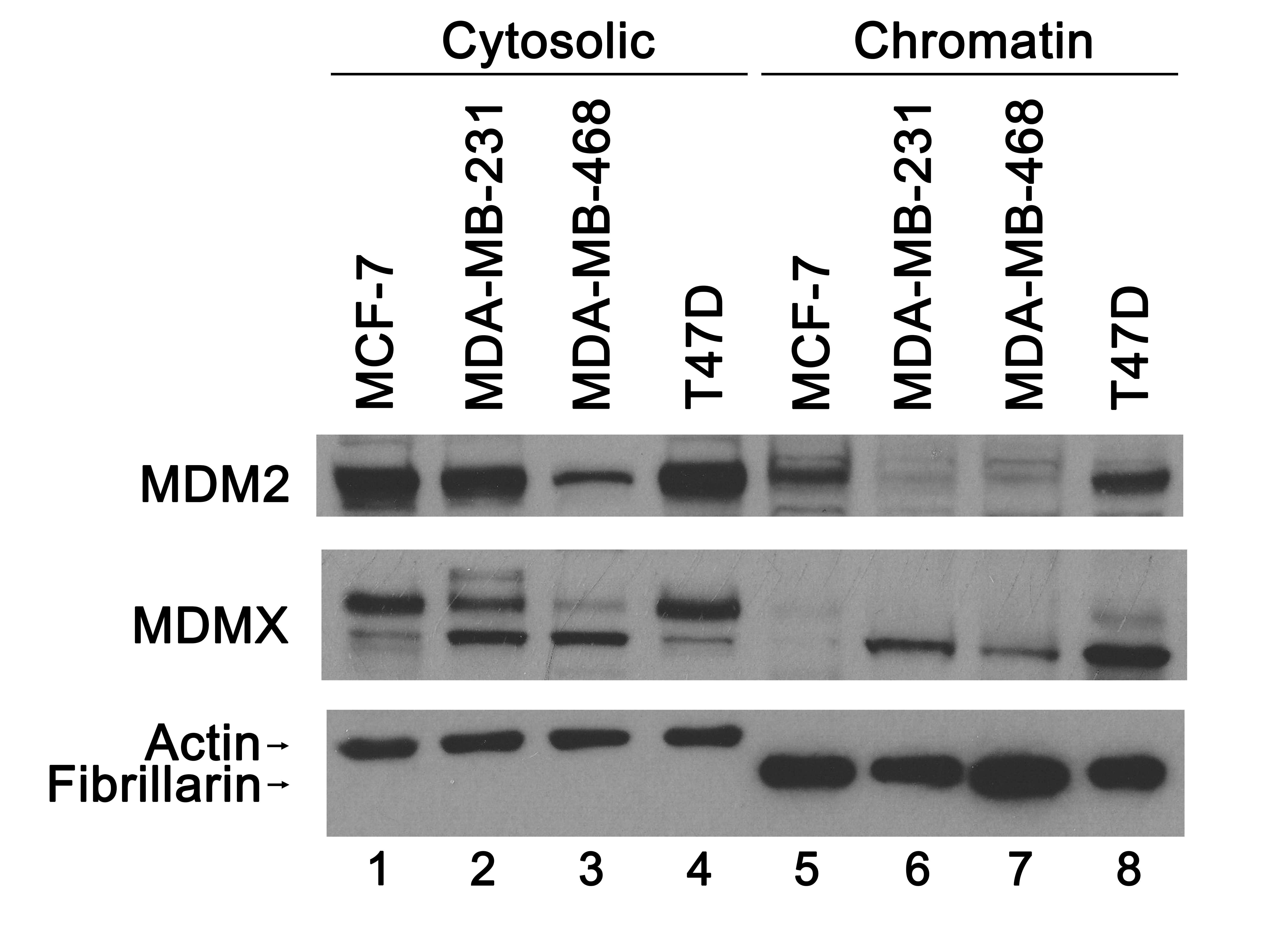

Supplement: Supplementary file 5 — Figure S5. Variable Levels of MDM2 and MDMX in Different Breast Cancer Cell Lines. Cell lysates from fractionated samples were analyzed to compare the relative levels of MDM2 and MDMX. Lanes 1–4 show cytoplasmic and 5–8 show chromatin proteins as indicated. Only ERα + cell lines MCF-7 and T47D showed high levels of chromatin localized MDM2 (this correlated with the activation of cell proliferation by MDM2). Fractionation was carried out as previously described [54]. (TIF 4685 kb) [file 13058_2018_1094_MOESM5_ESM.tif]
